# Supplementary material for: A 10-Minute “Mix and Read” Antibody Assay for SARS-CoV-2
Source: Viruses. 2021 Jan 20;13(2):143. doi: 10.3390/v13020143 (PMC7908974; doi:10.3390/v13020143)
Supplement: Supplementary file 1 [file viruses-13-00143-s001.pdf]

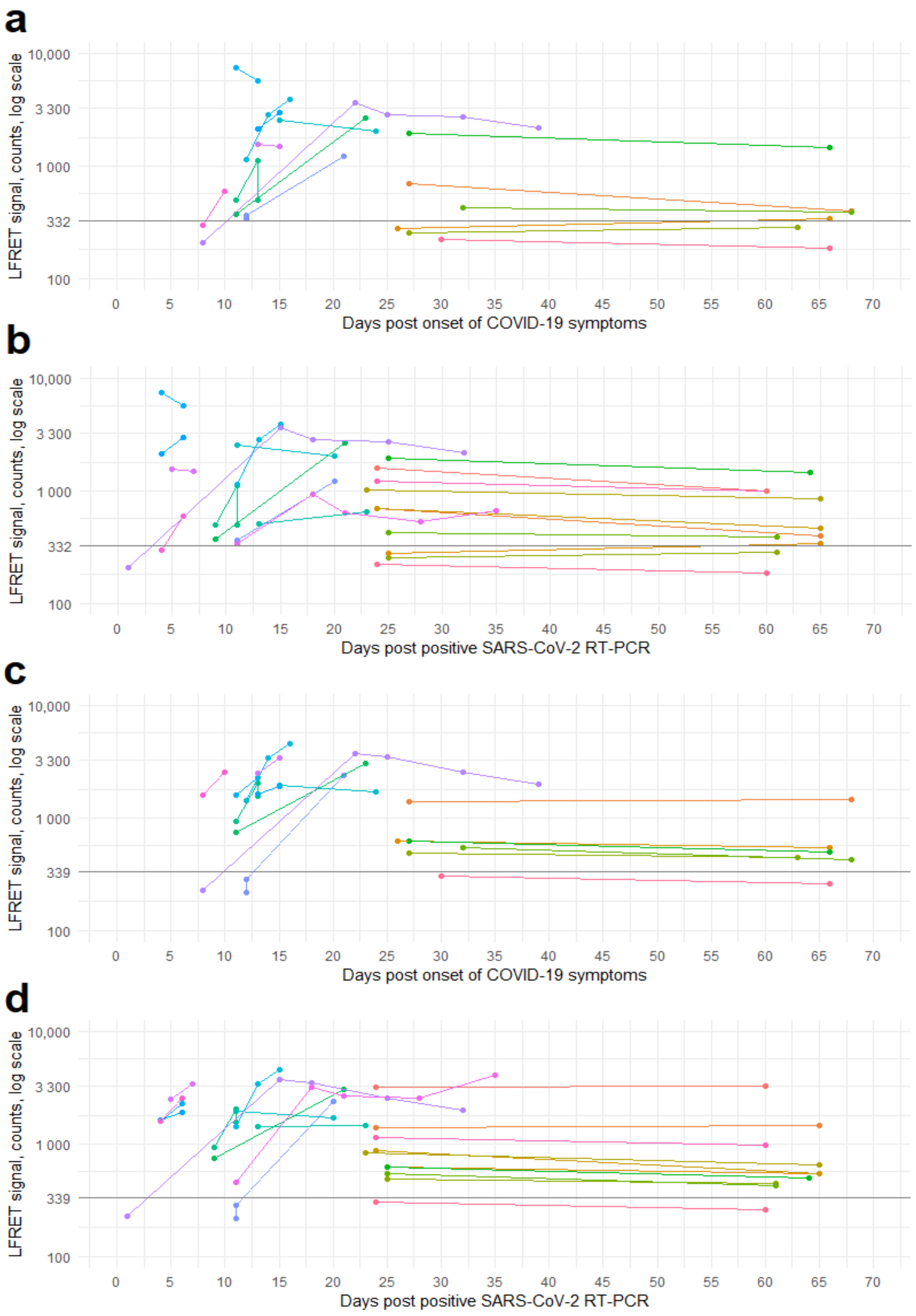

Figure S1. Development of LFRET signals in follow-up samples. (**a**) Days post onset of COVID-19 symptoms (*x*-axis) vs anti-NP LFRET signal (*y*-axis) (N = 38 samples from 16 individuals). (**b**) Days post positive SARS-CoV-2 PCR (x-axis) vs anti-NP LFRET signal (y-axis) (N = 53 samples from 22 individuals). (**c**) Days post onset of COVID-19 symptoms (x-axis) vs anti-SP LFRET signal (y-axis) (N = 38 samples from 16 individuals). (**d**) Days post positive SARS-CoV-2 PCR (*x*-axis) vs anti-SP LFRET signal (*y*-axis) (N = 53 samples from 22 individuals). Horizontal line is the LFRET cutoff. SP = spike glycoprotein. NP = nucleocapsid protein. LFRET = protein L–based time-resolved Förster resonance energy transfer

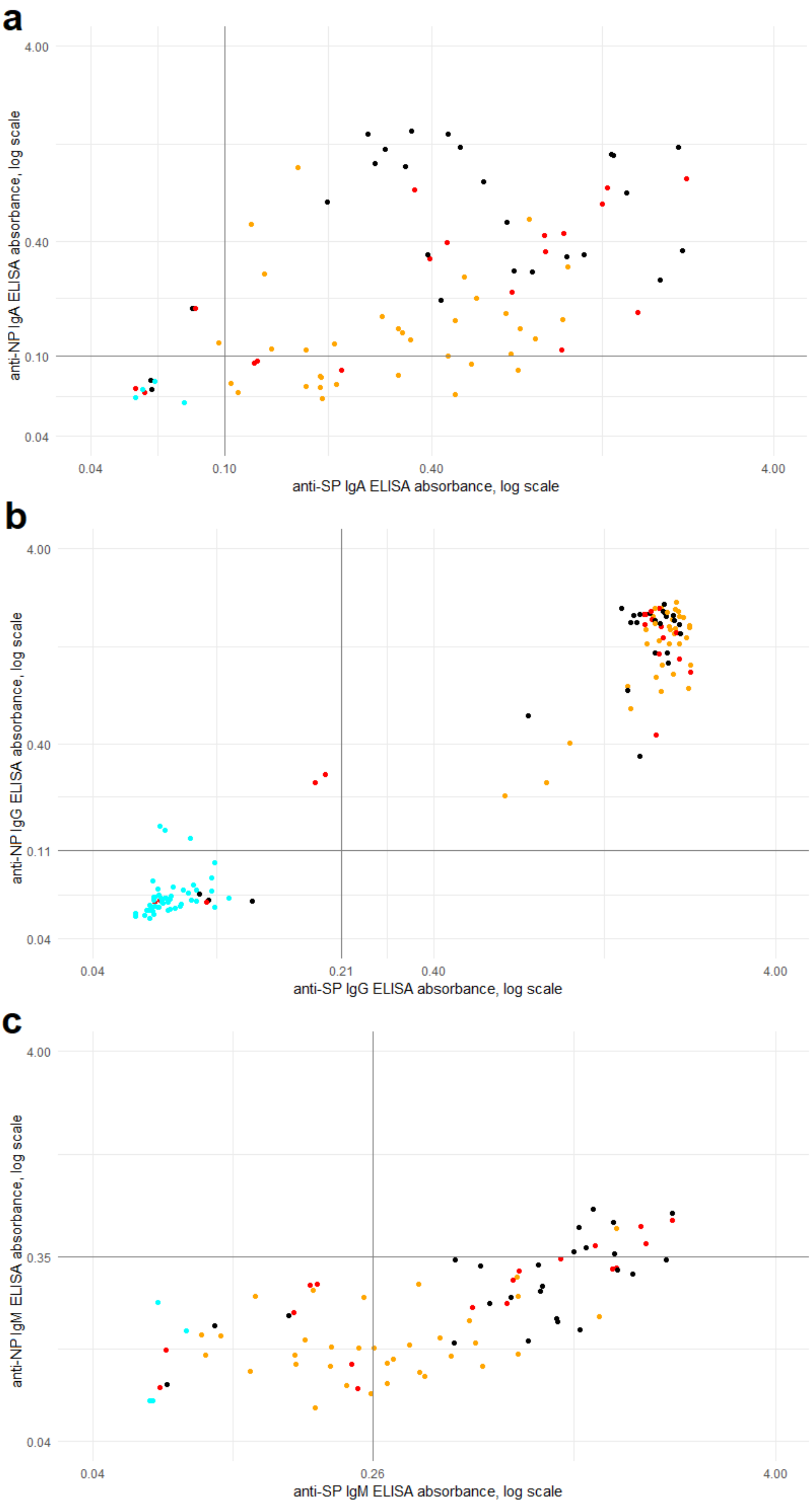

Figure S2. SARS-CoV-2 spike glycoprotein (SP) versus nucleoprotein (NP) ELISA. a) anti-SP vs. anti-NP IgA ELISA results (N=81, R=0.31) b) anti-SP vs. anti-NP IgG ELISA results (N=129, R=0.90). c) anti-SP vs. anti-NP IgM ELISA results (N=81, R=0.79). On the x-axis, anti-SP ELISA absorbance, on the y-axis anti-NP ELISA absorbance. Dot colours indicate SARS-CoV-2 PCR result and disease severity: cyan = PCR negative; yellow = non-hospitalized, PCR-positive; red = non-ICU hospitalized, PCR positive; black = hospitalized in ICU, PCR positive. Horizontal and vertical lines indicate ELISA cutoffs. R = Pearson's correlation coefficient.

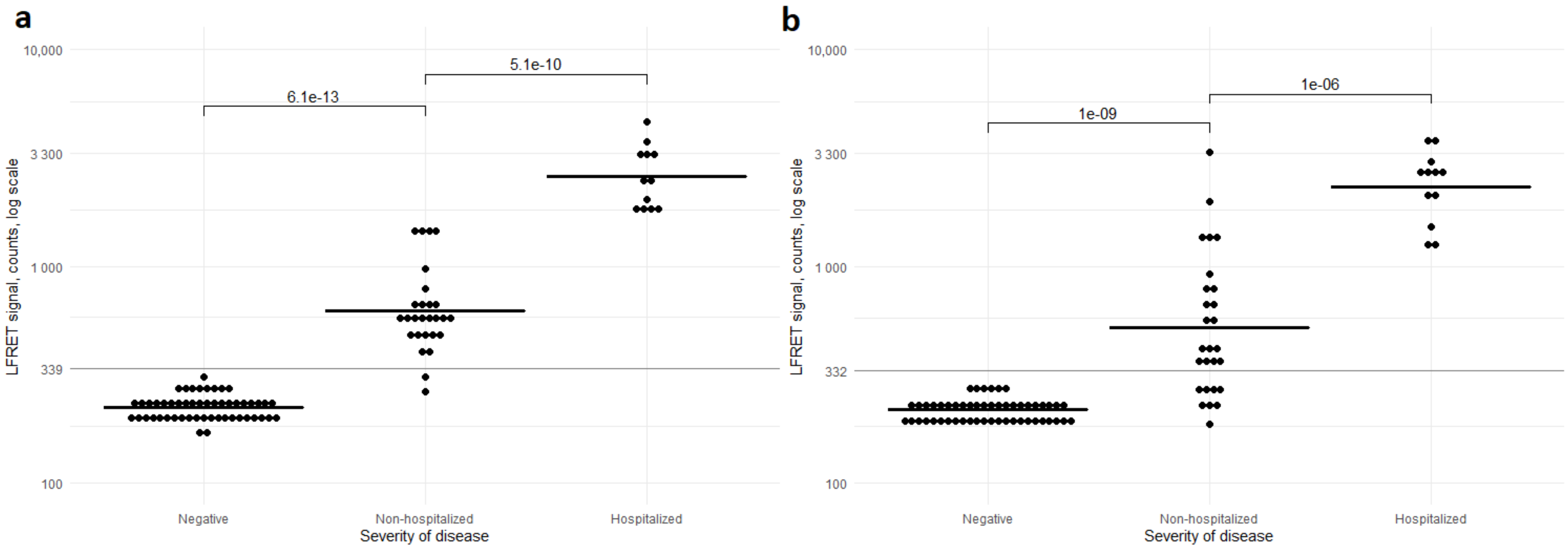

Figure S3. LFRET vs. disease severity after two weeks from onset. a) Severity (x-axis) vs. anti-SP LFRET (y-axis) b) Severity (x-axis) vs. anti-NP LFRET (y-axis). Negative = samples from comprehensively SARS-CoV-2 seronegative individuals (N=52). Non-hospitalized = samples from non-hospitalized COVID-19 patients (N=27). Samples from hospitalized patients (N=12) are grouped together to include samples from individuals treated outside of ICU (N=3) and in ICU (N=9). Horizontal lines indicate LFRET cutoffs (thin grey lines) and LFRET signal means (thick black lines). N.B, logarithmic y-axis scale. Statistical differences between groups, determined by Wilcoxon signed-rank test, are indicated by p values. SP = spike glycoprotein. NP = nucleoprotein. LFRET = protein L-based time-resolved Förster resonance energy transfer immunoassay.

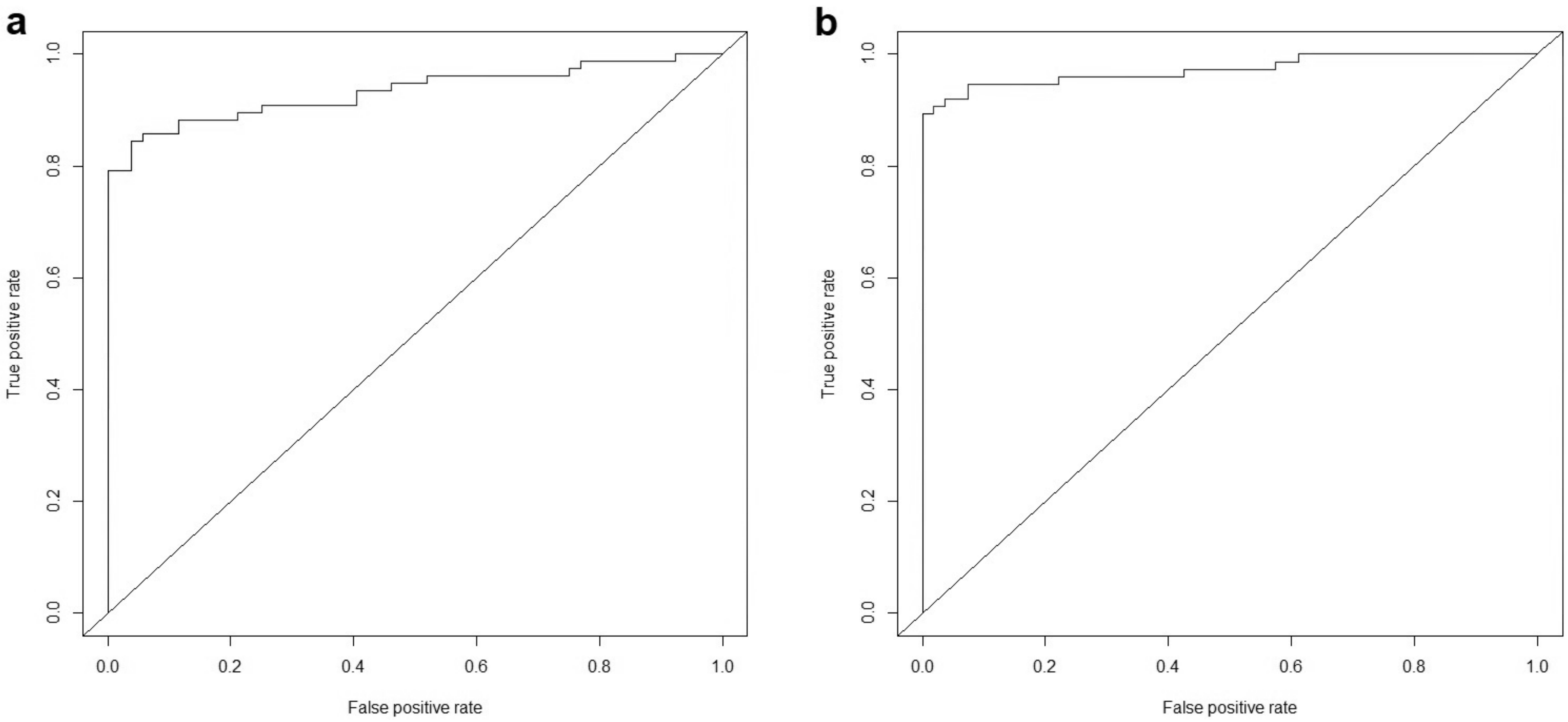

Figure S4. Receiver operating character (ROC) curves for LFRET. a) anti-NP LFRET. AUC = 0.94. b) anti-SP LFRET. AUC= 0.97. False positive rate on the x-axis, true positive rate on the y-axis. Enzyme immunoassay (IgA, IgG and IgM results combined) is used as reference. NP = nucleoprotein. SP = spike glycoprotein. LFRET = protein L–based time-resolved Förster resonance energy transfer immunoassay. Plotted in RStudio (v1.3.1073) with the ROCR library (v1.0-11).

Table S1. Agreement between ELISA and LFRET. Ig ELISA = IgA, IgG and IgM results combined: if any of these are positive, total Ig is considered positive. NP/SP ELISA/LFRET: if either NP or SP ELISA/LFRET is positive, the NP/SP result is considered positive. Number of samples in brackets. SP = spike glycoprotein. NP = nucleocapsid protein. LFRET = protein L–based time-resolved Förster resonance energy transfer immunoassay. ELISA = enzyme immunoassay.

	**anti-NP Ig ELISA**	**anti-NP IgG ELISA**	**anti-SP Ig ELISA**	**anti-SP IgG ELISA**	**anti NP/SP Ig ELISA**	**anti NP/SP IgG ELISA**
NP LFRET	88% (113/129)	89% (115/129)	91% (117/129)	90% (116/129)	87% (112/129)	89% (115/129)
SP LFRET	93% (120/129)	95% (122/129)	96% (124/129)	98% (127/129)	92% (119/129)	95% (122/129)
NP/SP LFRET	95% (122/129)	96% (124/129)	98% (126/129)	97% (125/129)	94% (121/129)	96% (124/129)

Table S2. Detailed information on samples with discordance between PCR, LFRET and ELISA results. Sample = sample id. NAT = nucleic acid testing result for SARS-CoV-2. SP = spike glycoprotein. NP = nucleocapsid protein. LFRET = protein L–based time-resolved Förster resonance energy transfer immunoassay. ELISA = enzyme immunoassay. MNT = microneutralization titer. + = result above cutoff. - = result below cutoff. Hosp. = Hospitalized, non-ICU. ICU = Hospitalized in ICU. Home = non-hospitalized. Neg. = no disease. NA = not available. .

Sample	NAT	Severity of disease	Days from onset of symptoms	Days from NAT	anti-SP LFRET	anti-NP LFRET	anti-SP ELISA IgA	anti-SP ELISA IgG	anti-SP ELISA IgM	anti-NP ELISA IgA	anti-NP ELISA IgG	anti-NP ELISA IgM	MNT
71	+	Hosp.	8	2	-	-	-	-	-	+	-	-	<20
65	+	ICU	8	1	-	-	-	-	-	-	-	-	<20
72	+	ICU	8	2	-	-	-	-	-	-	-	-	<20
70	+	ICU	13	4	-	-	-	-	-	+	-	-	20
7	+	Home	30	24	-	-	+	+	-	+	+	-	<20
24	+	Home	66	60	-	-	-	+	-	+	+	-	<20
86	+	Hosp.	NA	2	-	-	-	-	-	-	-	-	<20
82	-	Neg.	27	25	-	-	-	-	-	-	+	-	NA
92	-	Neg.	134	NA	-	-	-	-	-	-	+	-	<20
103	NA	Neg.	NA	NA	-	-	-	-	NA	NA	+	NA	<20
121	NA	Neg.	NA	NA	-	-	+	-	NA	NA	-	NA	<20

Table S3. Sensitivity and specificity of LFRET/ELISA in detecting microneutralization (MNT) positive (titer ≥ 20) samples. SP = spike glycoprotein. NP = nucleocapsid protein. LFRET = protein L–based time-resolved Förster resonance energy transfer immunoassay. ELISA = enzyme-linked immunosorbent assay. Only 15 MNT negative samples were tested in anti-NP IgA and IgM as well as anti-SP IgM ELISA, whereby these specificities warrant cautious interpretation.

	**anti-SP LFRET**	**anti-NP LFRET**	**anti-SP IgA ELISA**	**anti-SP IgG ELISA**	**anti-SP IgM ELISA**	**anti-NP IgA ELISA**	**anti-NP IgG ELISA**	**anti-NP IgM ELISA**
Sensitivity	98% (51/52)	90% (47/52)	98% (51/52)	98% (51/52)	79% (41/52)	87% (45/52)	98% (51/55)	17% (9/52)
Specificity	91% (50/55)	96% (53/55)	85% (47/55)	87% (48/55)	93% (14/15)	64% (10/15)	82% (45/55)	100% (15/15)

Table S4. Agreement between LFRET and commercial antibody assays. Euroimmun = Euroimmun SARS-CoV-2 ELISA (IgG). Abbott = Abbott Architect SARS-CoV-2 IgG. ELISA = enzyme-linked immunosorbent assay. SP = spike glycoprotein. NP = nucleocapsid protein. LFRET = protein L–based time-resolved Förster resonance energy transfer immunoassay.

	**anti-SP LFRET**	**anti-NP LFRET**
Euroimmun, seropositive samples	100% (27/27)	81% (22/27)
Euroimmun, seronegative samples	100% (48/48)	100% (48/48)
Euroimmun, all samples	100% (75/75)	93% (70/75)
Abbott, seropositive samples	100% (27/27)	93% (22/27)
